# Hospitalised neonates in Estonia commonly receive potentially harmful excipients

**DOI:** 10.1186/1471-2431-12-136

**Published:** 2012-08-29

**Authors:** Jana Lass, Kaisa Naelapää, Utpal Shah, Ruth Käär, Heili Varendi, Mark A Turner, Irja Lutsar

**Affiliations:** 1 Institute of Microbiology, Tartu University, Tartu, Estonia; 2 Pharmacy Department, Tartu University Hospital, Tartu, Estonia; 3 Department of Pharmaceutics and Analytical Chemistry, Faculty of Health and Medical Sciences, University of Copenhagen, Copenhagen, Denmark; 4 Cheshire, Merseyside & North Wales LRN, Medicines for Children Research Network, Alder Hey Children's NHS Foundation Trust, Liverpool, UK; 5 Tallinn Children’s Hospital, Tallinn, Estonia; 6 Children’s Clinic, Estonia Neonatal Unit, Tartu University Hospital, Tartu, Estonia; 7 University of Liverpool, Liverpool, UK; 8 Neonatal Unit, Liverpool Women’s Hospital, Liverpool, UK

**Keywords:** Harmful excipient, Neonate

## Abstract

**Background:**

Information on the neonatal exposure to excipients is limited. Our aim was to describe the extent of excipient intake by Estonian neonates; to classify the excipients according to potential neonatal toxicity and thereby to measure the extent of exposure of neonates to potentially harmful excipients.

**Methods:**

A prospective cohort study that recorded all medicines prescribed to patients aged below 28 days admitted to Tartu University Hospital from 01.02-01.08 2008 and to Tallinn Children’s Hospital from 01.02- 01.08 2009 was conducted. Excipients were identified from Summaries of Product Characteristics and classified according to toxicity following a literature review.

**Results:**

1961 prescriptions comprising 107 medicines were written for 348/490 neonates admitted. A total of 123 excipients were found in 1620 (83%) prescriptions and 93 (87%) medicines. 47 (38%) of these excipients were classified as potentially or known to be harmful to neonates. Most neonates (97%) received at least one medicine (median number 2) with potentially or known to be harmful excipient. Parabens were the most commonly used known to be harmful excipients and sodium metabisulphite the most commonly used potentially harmful excipient, received by 343 (99%) and 297 (85%) of treated neonates, respectively.

**Conclusions:**

Hospitalised neonates in Estonia are commonly receiving a wide range of excipients with their medication. Quantitative information about excipients should be made available to pharmacists and neonatologists helping them to take into account excipient issues when selecting medicines and to monitor for adverse effects if administration of medicines containing excipients is unavoidable.

## Background

Excipients are essential components of medicinal products required for manufacturing processes to assure several properties such as solubility, bioavailability and stability of the final dosage form. Concerns about the safety of pharmaceutical excipients are growing due to the increasing number of adverse reports, especially in neonates [1-4]. According to regulatory requirements, excipients have to be appropriately evaluated for safety [5-7]. Similar to active pharmaceutical ingredients, in most instances the safety data of excipients is based on adult exposure. Thus, information about their acceptability and safety in relation to the age and development status of the child is often lacking [8].

Neonates are the most vulnerable patient population when adverse reactions of excipients are considered. This is mainly due to organ immaturity and differences in pharmacokinetic (PK) and pharmacodynamic (PD) profiles compared to adults [9]. The inclusion of inadequately studied excipients in products used in neonates has resulted in tragedies such as the E-ferol incident where over 30 infants died after receiving an intravenous vitamin E product containing polysorbate 80 [10]. The potential for dose-related adverse reactions of excipients are of particular concern in preterm infants due to the immaturity of hepatic and renal functions [11,12].

The use of potentially toxic excipients in medicines given to neonates is not rare [13] as they are present in many commonly used drug products [14,15]. However, neonatal exposure to excipients is still poorly studied. Previous studies have been selective in terms of populations, indications or excipients. For example, the studies have included premature neonates only [13] or have been restricted to medicinal products used in gastroenterology [14] or to excipients known to be toxic (sodium benzoate, propylene glycol, methyl- and propyl parahydroxybenzoate, saccharin sodium, benzyl alcohol, benzalkonium chloride, polysorbate 80 and ethanol) [13,16]. We are not aware of studies looking at frequency of excipient use in an unselected cohort of hospitalised neonates.

Our objectives were first, to record the frequency of using potentially harmful excipients in hospitalised neonates; second, to classify the excipients used in neonatal wards in Estonia into categories according to the possible toxicity to neonates; and third, to describe how many neonatal prescriptions included potentially harmful excipients.

## Methods

This study used a cohort design recruiting in two centres over two non-overlapping but equivalent periods. A total of four wards in both paediatric hospitals of Estonia participated. All medicines prescribed to hospitalised neonates with postnatal age (PNA) below 28 days were recorded in Tartu University Hospital (TUH) from 1^st^ of February to 1^st^ of August 2008 and in Tallinn Children’s Hospital (TCH) from 1^st^ of February to 1^st^ of August 2009 [17]. The following information was extracted from the hospital records on twice weekly visits: demographic data (gestational age [GA], birth weight, gender, date of birth, PNA), admission and discharge dates and prescriptions for all medicinal products (International Nonproprietary and product names, formulations). The dose and frequency of medicines were not recorded for the purpose of this study and the use of standard intravenous replacement solutions, nutritional and technical products (including contrast agents), basic creams and ointments, parenteral nutrition solutions, vaccines and vitamins were excluded, although we acknowledge that some of these products may also contribute to the general excipient exposure.

The data from the neonatal wards at both hospitals were pooled and patients categorised based on the GA as preterm (<37 weeks) and full term (≥37 weeks) neonates.

The excipients present in the prescribed medicines were identified from their Estonian Summaries of Product Characteristics (SPC) and if the drug product was not registered in Estonia in September 2009, from the package insert leaflets. The excipients were divided into four categories as detailed in Table 1. The following literature sources were used for classification: Rowe’s Handbook of Pharmaceutical excipients 6^th^ ed. [18], European Commission guidelines on the excipients in the label and package leaflet of medicinal products for human use [19], European Medicines Agency (EMA) reflection paper formulations of choice for the paediatric population, 2006 [8], article by Fabiano *et al*. [20] and book from Costello *et al.*[21]. A PubMed database search was conducted by using the name of each excipient AND/OR synonyms AND “human toxicity” as search terms; no other limiters or terms were used to narrow or widen the search. If there were no results in the PubMed search or other abovementioned information sources, Google scholar ( http://scholar.google.com; last accessed 24^th^ September 2011) search was conducted using the same search terms.

**Table 1:** **Classification of excipients to which studied neonates were exposed according to available safety data **^**† **^

**Category**	**Safety status**	**Description**	**No of excipients identified**
**1**	Potentially safe	No adverse reactions reported	42
**2**	Potentially harmful and known to be harmful	Adverse reactions reported	47
**3**	No safety data found	No data found in the literature on human exposure and toxicity	19
**4**	Description of the excipient in SPC or PIL unspecific	Description does not allow a specific literature search	15
	**Total**		123

SPC- summary of product characteristics.

PIL - package insert leaflet.

^†^Based on data from Refs [8,18-21], and PubMed searches.

In this study, all excipients for which according to the abovementioned sources there were some safety concerns were classified as “potentially harmful” (Category 2 in Table 1).

The study was approved by the Ethics Review Committee of the University of Tartu. The study used anonymised data collected in routine clinical practice and did not require individual consent of the parent.

## Results

There were a total of 490 neonates (203 in TUH and 287 in TCH) hospitalised during the two study periods. Of these, 348 (176 preterm) received 1961 prescriptions for 107 medicines. Parenteral formulations were used most often (61/107) followed by manipulated oral solid formulations such as crushed tablets or opened capsules (19/107). Oral liquids (8/107), topical ointments and creams (6/107), ophthalmic (5/107), rectal (4/107) and inhalation (4/107) medicines were rarely used. In total 93 of 107 medicines (87%) and 1620 of 1961 prescriptions (83%) contained at least one excipient. The total number of different excipients was 123.

### Classification of excipients and neonatal exposure

One third of excipients (47/123) were classified as potentially harmful (Category 2, Table 1), including eight excipients already known to be harmful in neonates (Table 2). Another third of excipients (42/123) were classified as potentially safe (Category 1, Table 1). For the remaining 34 excipients human safety / toxicity data was not found in the literature (19/34; Category 3) or the description in the SPC was too unspecific to conduct a literature search (15/34; Category 4), Table 1. Many flavouring agents (e.g. banana and strawberry flavour) and essential oils (e.g. orange essential oil) had to be classified to the latter group as their chemical entity was not described in the SPC.

**Table 2:** Excipients identified as known to be harmful and potentially harmful and the prescribing prevalence of medications containing them in studied neonates

**Excipient**^**§**^	**No of medications containing / Total Number of prescriptions**	**Medications containing excipients (number of prescriptions**^**† **^**)**
**Known to be harmful to neonates**		
Parabens (methyl- and propyl parahydroxybenzoate)	6 / 343	Gentamicin inj (200), heparin inj (86), iron oral solution (32), heparin sodium ointment (12), prednisolone inj (7), cetirizine oral solution (6)
Saccharin sodium	6 / 173	Simeticone oral suspension (108), iron oral solution (32), zidovudine oral solution (17), cetirizine oral solution (6), miconazole ointment (6), sultamicilline powder for suspension (4)
Sodium benzoate	4 / 156	Simeticone oral suspension (108), caffeine solution (29), zidovudine oral solution (17), fludrocortisone inj (2)
Benzyl alcohol	6 / 125	Heparin inj (86), hydrocortisone inj (23), phenobarbital inj (13), clotrimazole ointment (1), diazepam rectal suspension (1), diclofenac inj (1)
Benzalkonium chloride	6 / 104	Salbutamol nebulisation solution (54), chloramphenicol ophthalmic solution (29), fusidic acid opthalmic solution (16), troxerutin gel (3), ipratropium bromide nebulisation solution (1), dexamethasone + neomycin + polymyxin B opthalmic solution (1)
Propylene glycol	7 / 79	Salbutamol nebulisation solution (54), phenobarbital inj (13), cetirizine oral solution (6), diazepam oral solution (3), ibuprofen tablet (1), diclofenac inj (1), diazepam rectal solution (1)
Polysorbate 80	4 / 70	Budesonide nebulisation solution (31), chloramphenicol ophalmic solution (29), miconazole ointment (6), tobramycin opthalmic ointment (4)
Ethanol	7 / 31	Heparin sodium ointment (12), miconazole ointment (6), dinoprostone inj (5), Lipase + amylase + protease tablet (3), diazepam oral solution (3), diosmectide powder for oral suspension (1), alprostadil inj (1)
**Potentially harmful excipients**		
Sodium metabisulphite	6 / 297	Gentamicin inj (200), dobutamine inj (45), epinephrine inj (36), morphine inj (14), diclofenac inj (1), norepinephrine inj (1)
Silica, colloidal anhydrous	10 / 195	Simeticone oral suspension (108), phenobarbital tablet (29), hyoscine butylbromide tablet (20), spironolactone tablet (18), furosemide tablet (6), ursodeoxycholic acid tablet (5), sotalol tablet (4), propranolol tablet (3), diosmectide powder for oral suspension (1), ibuprofen tablet (1)
Sorbic acid	2 / 168	Simeticone oral suspension (108), laurilsulphate + sorbitol + sodium citrate rectal solution (60)
Anhydrous sodium hydrogen phosphate (monobasic, dibasic)	10 / 122	Paracetamol suppository (29), paracetamol infusion solution (24), hydrocortisone powder for infusion solution (23), epoetin beta inj (22), dexamethazone inj (8), oxacillin powder for infusion solution (7), sultamicillin powder for infusion solution (4), ranitidine inj (2), pentoxifylline inj (2), ipratropium bromide nebulisation solution (1)
Sodium cyclamate	1 / 108	Simeticone oral suspension (108)
Disodium edetate	10 / 106	Budesonide nebulisation solution (31), piperacillin + tazobactam inj (25), fusidic acid ophthalmic solution (16), metoclopramide inj (10), dexamethazone inj (8), sulphametizole opthalmic solution (6), aminophylline inj (4), troxerutin ointment (3), esomeprazole inj (2), acetylcysteine inj (1)
Gelatin	5 / 51	Phenobarbital tablet (29), hydrochlorothiazide tablet (10), ursodeoxycholic acid tablet (5), digoxin tablet (4), lipase + amylase + protease tablet (3)
Sodium bicarbonate	4 / 44	Meropenem inj (32), povidone-iodine ointment (8), ceftazidime powder for infusion solution (3), enalapril tablet (1)
Boric acid	2 / 33	Chloramphenicol opthalmic solution (29), tobramycin opthalmic solution (4)
Borax	1 / 29	Chloramphenicol opthalmic solution (29)
Macrogols - polyethylene glycol	5 / 25	Povidone-iodine ointment (8), pentoxifylline tablet (7), Hydrocortisone + chlorhexidine ointment (4), lipase + amylase + protease tablet (3), propranolol tablet (3)
Trometamol	2 / 25	Ibuprofen inj (21), tobramycin ophthalmic ointment (4)
Glycine	1 / 22	Epoetin beta inj (22)
Calcium chloride dihydrate	1 / 22	Epoetin beta inj (22)
Leucine	1 / 22	Epoetin beta inj (22)
Titanium dioxide	5 / 21	Pentoxifylline tablet (7), ursodeoxycholic acid tablet (5), lipase + amylase + protease tablet (3), propranolol tablet (3), sildenafil tablet (3)
Povidone	4 / 19	Pentoxifylline tablet (7), furosemide tablet (6), drotaverine tablet (5), teophylline tablet (1)
Trolamine	2 / 15	Heparin sodium ointment (12), troxerutin ointment (3)
Cresol	1 / 11	Diosmectide powder for oral suspension (1)
Maltose	2 / 7	Tobramycin ophthalmic ointment (4), immunoglobuline inj (3)
Erythrosine	1 / 7	Pentoxifylline tablet (7)
Benzethonium chloride	1 / 7	Ketamine inj (7)
Sodium acetate trihydrates	2 / 7	Cetirizine oral solution (6), flecainide inj (1)
Cetostearyl alcohol	2 / 5	Hydrocortisone + chlorhexidine ointment (4), clotrimazole ointment (1)
Ethylendiamine	1 / 4	Aminophylline inj (4)
Macrogol cetostearyl ether	1 / 4	Hydrocortisone + chlorhexidine ointment (4)
Sodium laurilsulfate	1 / 3	Lipase + amylase + protease tablet (3)
Lactic acid	1 / 3	Milrinone inj (3)
Copovidone	1 / 3	Propranolol tablet (3)
Sodium formaldehyde sulfoxylate	2 / 2	Diazepam rectal solution (1), diclofenac inj (1)
Castor oil	1 / 1	Teophylline tablet (1)
Sorbitan stearate	1 / 1	Clotrimazole ointment (1)
Acacia	1 / 1	Diosmectide powder for oral suspension (1)

^**§**^European Pharmacopoeia names were used for excipients.

^**† **^ Pooled data from Tartu University Hospital (February - August 2008) and Tallinn Children’s Hospital (February - August 2009).

Almost all treated neonates (339/348; 97%) received medicines with at least one potentially harmful excipient and as many as 88% (307/348) received at least one of the eight excipients known to be harmful in neonates (Table 2). From the medicines prescribed, the median number of included excipients known to cause harm in neonates was two (interquartile range (IQR) 5–2; preterm neonates median of 3, range 1 to 15, IQR 4–2; term neonates median of 1, range 1 to 11, IQR 3–1).

### Potentially and known to be harmful excipients in medicines used in neonates

**Figure 1:**
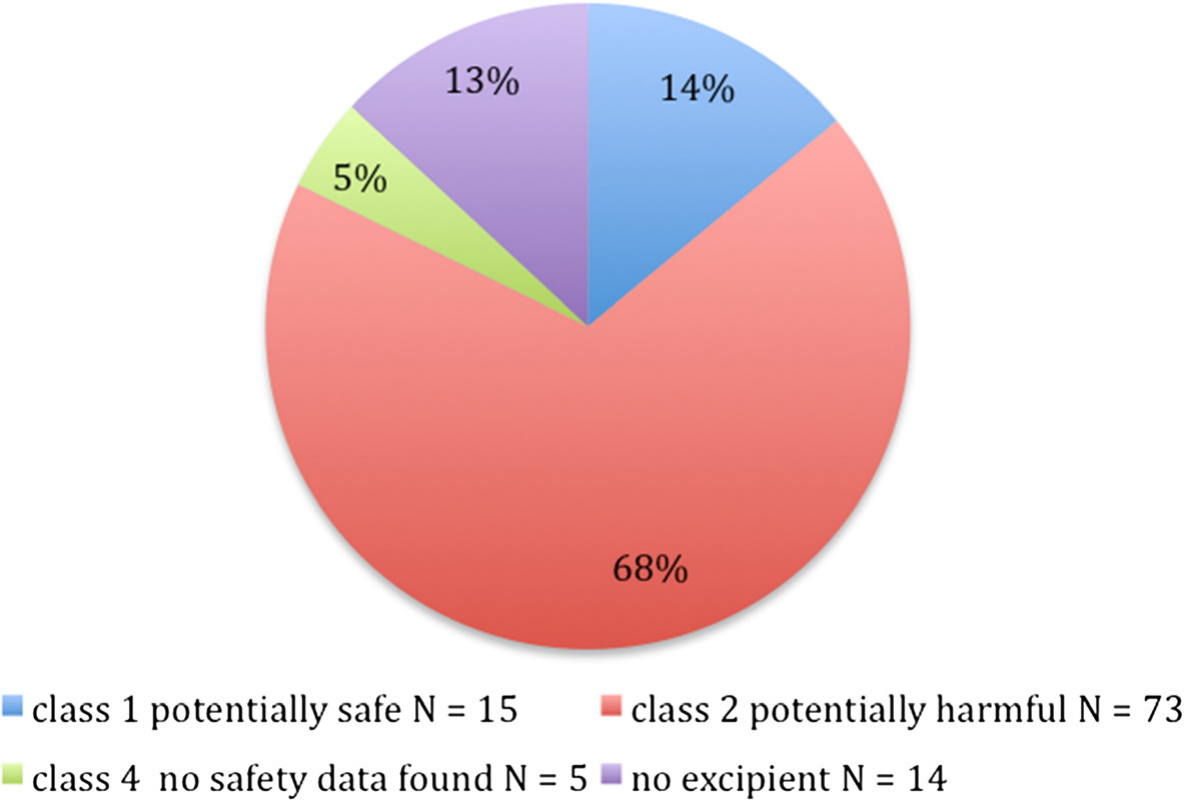
Proportion of prescribed medications, containing at least one excipient, in each safety category (every drug is listed once according to the worst-case scenario - into the most harmful category).

Figure 1 illustrates that approximately two thirds (73 /107, 68%) of all the medicines used in neonates contained at least one potentially harmful or known to be harmful excipient. The median number of such excipients per medicinal product was two and maximum was five (in simeticone oral suspension). The proportion of medicines containing potentially harmful excipients in preterm neonates was even higher than the general rate - 77%. When preterm and term neonates were compared in terms of medicines with potentially safe excipients the percentage of such medicines was the same in both groups (22%).

The detailed characteristics of excipients and their potential safety issues are described in Table 3.

**Table 3:** Excipients known to be harmful and potentially harmful to neonates used in study population, their applications and safety concerns

**Excipient**	**Functional category**^**† **^	**Applications and typical concentration ranges**^**† **^	**Safety concern**
**Known to be harmful to neonates**			
Parabens (methyl- and propyl parahydroxybenzoate)	Antimicrobial	Antimicrobial activity against yeasts and molds. Combination of Methyparaben (0.18%) and propylparaben (0.02%) for parenteral formulations. In combinations with propylene glycol (2-5%)/ imidurea	Hyperbilirubinemia in neonates. Irritant in injections / ophthalmic drugs. Hypersensitivity reactions. [18,19]
Saccharin sodium	Sweetening	0.02-0.5% w/w*	Urticaria with pruritus and photosensitivity reactions. [14]
Sodium benzoate	Antimicrobial, tablet / capsule lubricant	0.02-0.5% in oral medicines; 0.5% in parenteral medicines; 2-5% w/w tablet lubricant	Contact urticaria. [21] Topical irritant. Increased risk of hyperbilirubinaemia in neonates.
Benzyl alcohol	Antimicrobial, solvent	Up to 2% v/v* in parenteral/oral preparations, typically 1% v/v. 5% v/v and up used as solubilisers. 10% v/v local anaesthetic properties (parenterals, ophthalmic solutions, oitments)	Headache, vertigo, nausea, vomiting, diarrhea, metabolic acidosis, seizures, gasping. Hypersensitivity; fatal toxic syndrome in premature infants. Pain on injection, [8,18-20]
Benzalkonium chloride	Antimicrobial, antiseptic, solubilising, wetting	Ophthalmic preparations – preservative, 0.01-0.02% w/v*; In combination with other preservatives	Ototoxic when applied to ear, skin irritation and hypersensitivity Bronchoconstriction in asthmatics. Eye irritation. [18-20]
Propylene glycol	Antimicrobial, humectant, plasticizer, solvent, stabilizing, water-miscible cosolvent	Humectant – topical – approx.15%. Preservative –solutions / semisolids – 15-30%. Solvent or cosolvent: aerosol solutions 10-30%, oral solutions 10-25%, parenterals 10-60%, topical 5-80%	Skin irritation. Central nervous system (CNS) depression. High doses - cardiovascular, hepatic, respiratory adverse events. [18-20]
Polysorbate 80	Dispersing, emulsifying, non-ionic surfactant, solubilising, suspending, wetting	Emulsifying: alone in oil-in-water emulsions 1-15%; in combination 1-10%. To increase water-holding prop of ointments 1-10%. Solubilising: poorly soluble API*s in lipophilic bases 1-5%; insoluble APIs in lipophilic bases 0.1-3%	E-Ferol syndrome - thrombocytopenia, renal dysfunction, hepatomegaly, cholestasis, ascites, hypotension, metabolic acidosis. [18]
Ethanol	Solvent	In the USA, the max quantity of alcohol included in over the counter (OTC) medicines 0.5% v/v for products for use by children under 6 years of age. Parenteral products containing up to 50% of alcohol (e 95 or 96% v/v)	CNS depression - muscle incoordination, visual impairment. Negative synergic effects on CNS when associated with dextromethorfan. Chronic toxicity [8,18,20]
**Potentially harmful excipients**			
Sodium metabisulphite	Antimicrobial, antioxidant	Antioxidant in oral, parenteral, and topical formulations: 0.01–1.0% w/v, intramuscular 27% w/v. Antimicrobial: syrups.	Hypersensitivity. Paradoxical bronchospasm, wheezing, dyspnoe and chest tightness in asthmatic children. [18-20]
Colloidal anhydrous silica	Adsorbent; anticaking; emulsion stabilizer; glidant; suspending; tablet disintegrant; thermal stabilizer; viscosity-increasing	Improves flow properties of dry powders (0.1-0.5%) (tabletting); stabilizes emulsions (1.0-5.0%); thixotropic thickening/ suspending (2.0-10.0%); in aerosols to promote particulate suspension, eliminate hard settling, minimize clogging of spray nozzles (0.5-2.0%)	A possible sarcoidosis-inducing antigen [22]
Anhydrous sodium hydrogen phosphate (monobasic, dibasic)	Buffering; emulsifying; sequestering.	Buffering agent; sequestering agent. Concentrations are dependent on the formulation.	Gastrointestinal (GI) disturbances including diarrhea, nausea, and vomiting [18]
Sodium bicarbonate	Alkalizing; therapeutic.	To produce or maintain an alkaline pH in a preparation	Exacerbation of chronic heart failure in elderly [18]
Macrogols - polyethylene glycol	Ointment base; plasticizer; solvent; suppository base; tablet and capsule lubricant.	High molecular weight macrogols can be used as lubricants in tablet formulations; water solubility and bad penetration through skin makes them useful as ointment bases	Hypersensitivity reactions, hyperosmolarity, metabolic acidosis, and renal failure in burn patients. [18]
Trometamol	Buffering	Buffering agent, buffer range from 7.1–9	Hypersensitivity reactions. [23]
Cetostearyl alcohol	Emollient; emulsifying; viscosity-increasing.	Increasing viscosity; stabilizes emulsions; co-emulsifier; decreasing the amount of surfactant required	Hypersensitivity reactions. Contact dermatitis. [18,19]
Sodium lauryl sulphate	Anionic surfactant; detergent; emulsifying; skin penetrant; tablet and capsule lubricant, wetting	Tablet lubricant (1.0-2.0%)	Irritation to the skin, eyes, mucous membranes, upper respiratory tract, and stomach. [18]
Sorbitan stearate	Dispersing; emulsifying; nonionic surfactant; solubilizing; suspending; wetting	When used alone produces water-in-oil emulsions / microemulsions. In combination with polysorbate produces water-in-oil or oil-in-water emulsions / creams.	Hypersensitive reactions. [18]
Lactic acid	Acidulant	In injections in the form of lactate as a source of bicarbonate (0.012-1.16%)	Neonates have difficulty in metabolizing R-lactic acid, and this isomer and the racemate should therefore not be used in infants aged less than 3 months old. [18]
Sodium cyclamate	Sweetening	0.17% w/v as sweeter, in combination with saccharin	Photosensitivity. [18]
Disodium edetate	Chelating	Forms stable water-soluble complexes with alkaline earth and heavy-metal ions; concentrations 0.005-0.1%	Local inflammatory reactions. [18]
Gelatin	Coating; film-forming; gelling; suspending; tablet binder; viscosity-increasing	Tablet binder; microencapsulation	Local irritation. Hypersensitivity reactions, including serious anaphylactoid reactions [21]
Povidone	Disintegrant; dissolution enhancer; suspending; tablet binder	Binder in wet-granulation process; coating; solubilizer for poorly soluble drugs (0.5-5%)	Subcutaneous granulomas at the injection site. [21]
Trolamine	Alkalizing; emulsifying	When mixed in equimolar proportions with a fatty acid an emulsifying agent to produce fine-grained, stable oil-in-water emulsions will be formed (2-4%)	Hypersensitivity, skin irritant. [18]
Cresol	Antimicrobial preservative; disinfectant.	Antimicrobial preservative in parenteral formulations (0.15-0.3%)	Skin hypersensitivity reactions. [18]
Maltose	Sweetening; tablet diluent	Osmotic - ophthalmic drops and parenteral inf.	Single report of hyponatremia in a liver transplantation patient. [18]
Sorbic acid	Antimicrobial	As antimicrobial preservative (0.05-0.2%)	Irritant and allergic hypersensitivity skin reactions. [18,19]
Boric acid	Antimicrobial, buffering	As antimicrobial preservative in eye drops. Good buffering capacity to control pH.	Poisoning - abdominal pain, vomiting, diarrhea, erythematous rash, CNS depression. Convulsions, hyperpyrexia, and renal tubular damage. [18]
Borax	Alkalizing; antimicrobial; buffering; disinfectant; emulsifying; stabilizing	Antimicrobial preservative in eye preparations	Vomiting, diarrhea, erythema, CNS depression, and kidney damage. [18]
Glycine	Buffering; bulking; freeze-drying; tablet disintegrant; wetting	Cofreeze-dried excipient in injectable formulations	Disturbances of fluid and electrolyte balance; cardiovascular and pulmonary disorders. [18]
Calcium chloride dihydrate	Antimicrobial, water-absorbing.	Dehydrating properties	Stomach and heart disturbances. Eye irritant, dermatitis. [18]
Leucine	Antiadherent; flavoring; lubricant	As antiadherent to improve the deagglomeration	Moderately toxic by the s/c route. [18]
Titanium dioxide	Coating as opacifier, pigment	As a white pigment and opacifier	Possibly carcinogenic [24]
Benzethonium chloride	Surfactant, antiseptic, wetting and/or solubilizing	As an antimicrobial preservative (0.01-0.02% w/v)	Probably neurotoxic [25]
Erythrosine	Cherry-pink/red synthetic coal tar dye	Dye	Toxic to human lymphocytes in vitro, binds directly to DNA. [26]
Sodium cetate trihydrate	Antimicrobial; buffering; flavoring, stabilizing	As a buffering agent and as an antimicrobial preservative	Poisonous if injected i/v, an irritant to the skin and eyes. [18]
Ethylendiamine	Counter ion	Counter ion of theophylline	Hypersensitivity reactions [27]
Macrogol cetostearyl ether	Emulsifying; penetration enhancer; solubilizing; wetting	Solubilizing agent, enhancing effect on the skin permeation	Moderately toxic. [18]
Copovidone	Film-forming; granulation aid; tablet binder	As a film-forming agent (0.5-5%); tablet binder (direct compression and wet granulation) (2.0-5.0%)	Moderately toxic by ingestion, gastric disturbances. [18]
Sodium formaldehyde sulfoxylate	Antioxidant	Antioxidant in parental, rectal solutions	Moderately toxic by ingestion, when heated to decomposition it emits toxic fumes. [18]
Castor oil	Emollient; oleaginous vehicle; solvent.	extended release agent	Contact dermatitis. [18]
Acacia	Emulsifying; stabilizing; suspending; tablet binder; viscosity-increasing.	Viscosity increasing agent (as it is in powder for oral suspensions)	Hypersensitivity reactions. [18]

* w/w – weight in weight.

v/v – volume in volume.

w/v – weight in volume.

API – active pharmacutical ingredient.

^**† **^ Source: Ref [18].

The most common excipients that are known to cause harm were propylene glycol and ethanol, both present in seven products. In relation to prescription frequency, the most common excipients known to cause harm were parabens (methyl- and propylparahydroxybenzoate) used as preservatives in parenteral gentamicin given to 57% of treated neonates. The gentamicin product also included another potentially harmful excipient, namely sodium metabisulphite (Table 4).

**Table 4:** Most commonly prescribed medicines (received by >10 patients) containing known to be harmful or potentially harmful excipients

**Rank**	**Active substance, drug formulation**	**No of prescriptions**	**Potentially harmful or known to be harmful excipients**
1	Gentamicin, inj solution	200	Parabens, sodium metabisulphite
2	Simeticone, oral suspension	108	Sodium benzoate, saccharin sodium, silicium dioxide, sodium cyclamate, sorbic acid
3	Heparin, inj solution	86	Benzyl alcohol, parabens
4	Laurilsulphate + Sorbitol + Sodium citrate, rectal solution	60	Sorbic acid
5	Salbutamol, nebulisation solution	54	Benzalkonium chloride, propylene glycol
6	Dobutamine, inj solution	45	Sodium metabisulphite
7	Epinephrine, inj solution	36	Sodium metabisulphite
8	Iron, oral solution	32	Parabens, saccharin sodium
9	Budesonide, nebulisation solution	31	Polysorbate 80, disodiumedetate
10	Chloramphenicol, opthalmic solution	29	Benzalkonium chloride, polysorbate 80, borax, boric acid
11	Caffeine, solution	29	Sodium benzoate
12	Phenobarbital, tablet	29	Silicium dioxide, gelatin
13	Paracetamol, suppository	29	Disodium hydrogen phosphate
14	Piperacillin + tazobactam, inj solution	25	Disodium edetate
15	Paracetamol, inj solution	24	Disodium hydrogen phosphate
16	Hydrocortisone, inj solution	23	Benzyl alcohol, disodium hydrogen phosphate
17	Epoetin beta, inj solution	22	Disodium hydrogen phosphate, glycine, calcium chloride dihydrate, leucine,
18	Ibuprofen, inj solution	21	Trometamol
19	Hyoscine butylbromide, tablet	20	Silicium dioxide
20	Spironolactone, tablet	18	Silicium dioxide
21	Zidovudine, oral solution	17	Sodium benzoate, saccharin sodium
22	Fusidic acid, ophthalmic solution	16	Benzalkonium chloride, disodium edetate
23	Morphine, inj solution	14	Sodium metabisulphite
24	Phenobarbital, inj solution	13	Benzyl alcohol, propylene glycol
25	Heparin sodium, topical gel	12	Parabens, ethanol, trietanolamine,
26	Insulin, inj solution	11	Cresol

Inj - injection.

Simeticone oral suspension was the second most commonly prescibed medicine, given to 31% of neonates. The simeticone product contained two excipients known to cause harm – saccharin sodium and sodium benzoate, and also three other potentially harmful excipients - colloidal anhydrous silica, sorbic acid and sodium cyclamate (Table 4).

Of excipients known to cause harm as an excipient in older age groups, but where there is no neonatal data, colloidal anhydrous silica and anhydrous sodium hydrogen phosphate were most commonly present, both in 10 medicines. Medicines containing sodium metabisulfite were prescribed most frequently in this category. Again its frequent exposure was driven by wide use of parenteral gentamicin.

Approximately two thirds of parenterally used products (29/47) contained some potentially harmful excipient. The situation was even worse for other formulations - all of the prescribed rectal, topical, inhalation, oral solutions and oral suspensions contained at least one potentially harmful excipient. The use of topical agents in neonates was rare and only 8/33 products contained excipient known to be toxic to neonates. Only one of the 19 orally administered solid drug formulations and one of the five ocular formulations were free of potentially harmful excipients. Not surprisingly, most of medicines free from potentially harmful excipients were parenteral single-dose antibacterial or antifungal formulations (13/ of all the 35 medicines without harmful excipients).

A total of 19 medicines were licensed for use in neonates (6 for preterm and all for term neonates). Approximately half of them (3/6 in preterm and 8/19 in term neonates) contained at least one potentially harmful excipient.

The amount of the excipients in the drug formulation was present in the SPCs for only two medications (metoclopramide injection solution and esomeprazole powder for injection).

## Discussion

To the best of our knowledge this is the first study looking at the excipients in medicines given to an unselected group of hospitalised neonates. We describe the presence of over a hundred different excipients and demonstrate that almost all drugs used in neonates (including those licensed for neonates) contain at least one potentially harmful excipient. The safety of the majority of these excipients in neonates is not easily assessable as the information in the SPCs is scarce and published studies on the topic are rare. The fact that over a third of excipients present in medicines are known or suggested to be potentially harmful to neonates is even more worrisome.

As we are not aware of any validated classification of excipients in terms of neonatal safety and tolerability, we created a tentative classification for the study purposes in which all administered excipients were divided into the categories by conducting a literature search. As a result we show, that in addition to excipients known to cause harm (e.g. propylene glycol, ethanol etc.), many other excipients (e.g. sodium metabisulfite and colloidal anhydrous silica) could possess some safety concerns to neonates as they have been found to be harmful in older children and adults (Table 3).

The proportion of medicines containing potentially harmful excipients in our study (68%) is higher than the one recently published by van Riet-Nales *et al.*[28] in the Netherlands (52% of oral liquid formulations and 7% of all parenteral products). This difference is most likely explained by the methodological variations, regional characteristics in marketed product ranges and by differences in classifying excipients into the toxicity categories. In the Dutch study only “known to be toxic” excipients were taken into the analysis while in our study a very conservative approach was taken and the excipients were classified into the “potentially harmful” category even if only some data on human toxicity had been published (also when used as a substance) as one could not assure that the same agent does not cause any harm when used in small quantities as an excipient. However, we declare that all classifications without clear data from appropriately designed studies are hypothetical.

The use of excipients is unavoidable. Some can be found in food and WHO has set an acceptable daily intake for several of these. Nevertheless the amount of excipients in drug formulations is rarely reported in the SPCs making the evaluation of precise exposure and assessment of true harmfulness of these substances to neonates impossible. Investigators have requested information about exact excipient amounts from the manufacturers [13], but this is very time-consuming and does not allow a large number of medicines to be investigated. Whilst the FDA provides an online database of excipient concentrations in licensed medicines, it does not identify the marketed medicinal products for confidentiality reasons ( http://www.accessdata.fda.gov/scripts/cder/iig/getiigWEB.cfm; last accessed 14^th^ January 2012). The limits on data about excipients are exemplified by the 24 products (22% of the total used), which were not officially registered in Estonia or through the centralised EU mutual recognition agreement. These medicines were imported to Estonia according to the *ad hoc* approval of the Estonian State Agency of Medicines. The list of these drugs included parenteral phenobarbital, petidine and sodium oxybutyrate. The SPCs of most of these products could not be accessed, as they did not have European SPCs. The lack of quantitative data despite considerable effort to obtain information from the manufacturers means that our results may underestimate the extent of excipient exposure in these patients. However, this does not affect our conclusions. We suggest that quantitative information about high-risk excipients should be made available to pharmacists and physicians working with neonates.

Despite the lack of quantitative information, it could be that even if the excipient is known to be harmful, the daily intake will not exceed the toxic threshold due to the small quantities used in drug formulations. For example, from using a parenteral gentamicin product, a premature infant weighting 500 g and receiving a daily dose of 2 mg gets a maximum of 0.1mcg of parabens (methyl- and propylparahydroxybenzoate, parenteral formulations contain up to 0.75% parabens). When comparing this value to the allowed daily intake of 10 mg/kg body weight in adults it is obvious that the quantities are far below the toxic threshold. However, the fact that in neonates organs and thus the PK pathways are not fully matured may change the situation drastically [20]. Furthermore Allegaert *et al*. showed recently that a short duration of unintended propylene glycol administration at a median dose of 34 mg/kg over 48 hours was well tolerated by (pre)term neonates [29]. At the same time the authors stress that long-term safety of propylene glycol is still not established. We believe that the well-known toxic or potentially harmful excipients need careful safety assessment and determination of PK/PD profiles in neonates.

It has been stated that certain colouring and flavouring agents such as erythrosine (cherry-pink dye) need to be avoided in children [30]. This study also found that the SPC description of 15 excipients was too unspecific to allow a safety evaluation. Many of these excipients were flavouring agents such as banana flavour, cherry aroma, strawberry, raspberry flavour etc. Without further information in the SPC the safety assessment and critical evaluation of the administration of these agents to neonates is impossible in clinical practice.

We found that the formulation of the most commonly used drug in Estonian neonates, gentamicin, contains well-known harmful excipients – parabens. However a paraben-free gentamicin product is also registered in Europe. With this our data are in line with van Riet-Nales *et al.*[28] who also showed that for 22% of oral liquid paediatric medicines contain potentially harmful excipients an alternative formulation lacking such excipient was available with the same active chemical entity. This indicates that health professionals have a low awareness on safety of excipients. We recommend that when compiling a formulary for the treatment of vulnerable patients such as neonates attention should also be paid to the identification of excipients in medicines.

### Limitations

The most important limitation of our study is the lack of information on the exact amounts of excipients in medicines which precludes us making any conclusions on the real excipient exposure. This limitation was beyond our control because manufacturers do not disseminate this information. The other challenges are the use of a novel and non-validated classification system and restriction of the study to one country only. Also we did not collect information about dosage regimens since this would have been uninterpretable in the absence of quantitative information about the excipient content of the prescribed medicines. Another issue, possibly characteristic of other small markets, was the significant use (22%) of unlicensed medicines and thus unavailability of SPCs. In these cases the excipient content was recorded according to the package insert leaflets. Altough we appreciate that only excipients of intravenous, topical and opthalmic products and known to be toxic excipients have to be declared in the package leaflet, we assume that this will not significally affect our conclusions. Finally the study was conducted in a small country and thus these results cannot be generalised to other countries. These limitations do not undermine our findings that neonates are frequently exposed to a range of potentially harmful and known to be toxic excipients. Many of these limitations will be addressed in a recently commenced international research project, the European Study of Neonatal Excipient Exposure (ESNEE) [31], which evaluates the neonatal excipient exposure of over 20 European countries. The aims of the project are to show which excipients are used in formulations given to neonates treated in European hospitals, to conduct a review of toxicological studies on excipients and to investigate excipient kinetics during treatment with commonly prescribed neonatal medicines.

## Conclusion

Hospitalised neonates commonly receive numerous excipients, several of which have not until now been discussed regarding their potential harm to neonates. There is an urgent need for the careful toxicological assessment of excipients as the information in the published literature is limited. Information about excipients should be made available to pharmacists and neonatologists to assist the selection of the most appropriate medicines for neonates. When excipients cannot be avoided, professionals should have access to quantitative and qualitative information that allows them to assess risk and monitor vulnerable patients appropriately.

## Competing interests

None of the authors declares any competing interest.

## Authors’ contributions

JL, RK and HV carried out the data collection. JL and IL participated in the design of the study. JL and IL performed the analysis. KN, MT, US and IL drafted the manuscript. All authors read and approved the final manuscript.

## Pre-publication history

The pre-publication history for this paper can be accessed here:

http://www.biomedcentral.com/1471-2431/12/136/prepub
